# Влияние нарушений углеводного обмена на ранние и отдаленные клинические исходы у пациентов с COVID-19 по данным регистров АКТИВ и АКТИВ 2

**DOI:** 10.14341/probl13175

**Published:** 2023-02-25

**Authors:** В. В. Салухов, Г. П. Арутюнов, Е. И. Тарловская, Т. И. Батлук, Р. А. Башкинов, И. В. Самусь, Е. С. Мельников, М. А. Трубникова, А. Г. Арутюнов

**Affiliations:** Военно-медицинская академия им. С.М. Кирова; Евразийская Ассоциация Терапевтов; Российский национальный исследовательский медицинский университет им. Н.И. Пирогова; Евразийская Ассоциация Терапевтов; Приволжский исследовательский медицинский университет; Евразийская Ассоциация Терапевтов; Евразийская Ассоциация Терапевтов; Северо-Западный государственный медицинский университет имени И.И. Мечникова; Кузбасская клиническая психиатрическая больница; Евразийская Ассоциация Терапевтов; Северо-Западный государственный медицинский университет имени И.И. Мечникова; Евразийская Ассоциация Терапевтов; «Фрезениус Медиал Кеа Кубань»; Евразийская Ассоциация Терапевтов; Национальный институт здравоохранения им. академика С. Авдалбекяна

**Keywords:** СOVID-19, сахарный диабет 2-го типа, гипергликемия, летальность, предикторы

## Abstract

**ОБОСНОВАНИЕ:**

ОБОСНОВАНИЕ. Многочисленные исследования свидетельствуют о высокой встречаемости различных нарушений углеводного обмена (НУО) при новой коронавирусной инфекции (НКИ), утяжеляющих ее течение и приводящих к большей частоте смертельных исходов. Это актуализирует поиск факторов риска неблагоприятных исходов и оценку отдаленных последствий COVID-19 у пациентов с НУО.

**ЦЕЛЬ:**

ЦЕЛЬ. Изучить взаимосвязь НУО у пациентов с COVID-19 с летальностью, течением инфекции и отдаленными последствиями, а также выявить факторы риска неблагоприятного течения заболевания.

**МАТЕРИАЛЫ И МЕТОДЫ:**

МАТЕРИАЛЫ И МЕТОДЫ. Выполнен ретроспективный анализ данных объединенных многоцентровых неинтервенционных регистров реальной клинической практики АКТИВ и АКТИВ 2, включивший 9290 пациентов с COVID-19 различной степени тяжести, перенесенной в период с 29.06.2020 г. по 29.11.2020 г (АКТИВ) и с 01.10.2020 г. по 30.03.2021 г. (АКТИВ 2). Пациентов разделяли на группы: группа 1 — пациенты без НУО, n=6606, группа 2 — пациенты с впервые выявленной гипергликемией (ВВГ), n=1073, группа 3 — лица с сахарным диабетом 2 типа (СД2) в анамнезе, n=1611. В группах оценивали клинико-лабораторные показатели, наличие сопутствующей патологии и летальность в период инфекции, а также через 12 мес — состояние углеводного обмена пациентов и их самочувствие.

**РЕЗУЛЬТАТЫ:**

РЕЗУЛЬТАТЫ. Распространенность НУО составила 28,9% случаев, из которых 17,3% — СД2, а 11,6% случаев представлены ВВГ. Летальность пациентов с гипергликемией любого генеза составила 10,6% случаев, что значимо выше по сравнению с пациентами без таковой (3,9%), шансы наступления летального исхода у больных с СД2 увеличивались в 2,48 раза, а в группе пациентов с ВВГ — в 2,04 раза, у пациентов без НУО летальность, напротив, уменьшалась в 2,94 раза. Через 12 мес у пациентов с НУО выявлено значимо большее количество жалоб с преобладанием их у пациентов с СД2. Спустя год после инфекции в группе лиц с ВВГ только 1,7% имели СД2 и получали пероральные сахароснижающие препараты. Разработанная прогностическая модель определения риска развития летального исхода основывается на выявленных предикторах: сопутствующая ишемическая болезнь сердца, инфаркт миокарда или инсульт в анамнезе, более высокая гликемия и старший возраст.

**ЗАКЛЮЧЕНИЕ:**

ЗАКЛЮЧЕНИЕ. НУО приводят к ухудшению течения НКИ, большему количеству смертельных исходов. Через год после инфекции у пациентов с СД2 и ВВГ чаще сохраняются жалобы, а ВВГ в большинстве случаев после инфекции нивелируется.

## Abstract (en)

**BACKGROUND:**

BACKGROUND: Numerous studies indicate a high incidence of various disorders of carbohydrate metabolism against the new coronavirus infection. These disorders aggravate the course of infection and increase mortality. Thereby, analysis of risk factors for unfavorable outcomes and assessment of the long-term consequences of COVID-19 in patients with impaired carbohydrate metabolism is of great importance.

**AIM:**

AIM: To investigate the association between carbohydrate metabolism disorders in COVID-19 patients and mortality, course of infection, long-term consequences, as well as to identify risk factors for an unfavorable disease course.

**MATERIALS AND METHODS:**

MATERIALS AND METHODS: A retrospective analysis of data from the combined multicenter non-interventional real-world AKTIV and AKTIV 2 registries was performed. The sample included 9290 patients who had COVID-19 with varying severity from June 29, 2020, to November 29, 2020 (AKTIV) and from October 01, 2020, to March 30, 2021 (AKTIV 2). The patients were divided into 3 groups: Group 1 — patients with intact carbohydrate metabolism, n=6606; Group 2 — patients with newly diagnosed hyperglycemia (NDH), n=1073; Group 3 — patients with a history of type 2 diabetes mellitus (DM2), n=1611. The groups were assessed for clinical and laboratory parameters, comorbidities, mortality, carbohydrate metabolic status, and well-being during the infection and at 12 months.

**RESULTS:**

RESULTS: The prevalence of carbohydrate metabolism disorders (CMD) was 28,9%, with DM2 patients accounting for 17,3% and patients with newly diagnosed hyperglycemia (NDH) for 11,6%. The mortality rate of patients with hyperglycemia of any origin was 10.6%, which was significantly higher compared to patients without hyperglycemia (3,9%). The probability of lethal outcome increased 2,48-fold in the group of patients with DM2 and 2,04-fold in the group of patients with NDH. At the same time, the probability of a lethal outcome decreased 2,94-fold in patients without CMD. At 12 months, patients with CMD showed a significantly higher frequency and longer persistence of complaints. This trend was more pronounced in patients with DM2 than in those with NDH. Only 1,7% of patients from the NDH group had type 2 diabetes and were receiving oral hypoglycemic medications one year after the infection. A prognostic model was developed to determine the risk of lethal outcome. The model included such known predictors as concomitant ischemic heart disease, history of myocardial infarction or stroke, blood glucose level, and age.

**CONCLUSION:**

CONCLUSION: Carbohydrate metabolism disorders aggravate the course of COVID-19 and increase mortality. One year after infection, patients with DM2 and NDH were more likely to have symptoms typical for post-COVID syndrome, and NDH resolved in most cases after the infection.

## ОБОСНОВАНИЕ

С конца 2019 г. новая коронавирусная инфекция (НКИ), объявленная Всемирной организацией здравоохранения пандемией, стремительно распространилась по всему миру, определив чрезвычайно высокие заболеваемость и смертность. В большом количестве исследований продемонстрировано, что лица, страдающие хроническими кардиометаболическими заболеваниями, характеризуются более тяжелым течением и неблагоприятным прогнозом в период COVID-19 и в отдаленные сроки после инфекции [1–3].

К числу таких заболеваний, которые могут повлиять на течение и исходы инфекции, вызванной SARS-CoV-2, относится сахарный диабет (СД), представляющий собой часто встречаемую патологию и характеризующийся стабильным ростом распространенности в нашей стране и в мире [4]. Общая численность больных СД в Российской Федерации на 01.01.2022 г. составила 4 871 863, из них с СД 2 типа (СД2) — 92,3% (4,5 млн). Однако если учесть долю недиагностированного СД2 в России, которая в среднем оценивается в 54%, можно заключить, что реальное количество больных должно составлять не менее 10 млн человек (6,9% населения РФ) [5].

Многочисленные исследования показали, что пациенты с COVID-19 и СД в большей степени нуждаются в госпитализации и проведении неинвазивной кислородотерапии, чаще требуют перевода в отделение интенсивной терапии, проведения искусственной вентиляции легких и характеризуются более высокой летальностью по сравнению с больными без нарушений углеводного обмена (НУО) [6][7]. Наряду с этим в реальной клинической практике и по данным публикаций отмечается весьма значительная доля пациентов (~10–60%), у которых в условиях стационара диагностируется впервые выявленная гипергликемия (ВВГ). ВВГ в постковидном периоде может как нивелироваться, так и трансформироваться в СД, причины и патогенез которого еще предстоит исследовать [8–10].

Учитывая высокую распространенность НУО и существующие данные о взаимном влиянии НУО и COVID-19, представляется чрезвычайно важным сфокусироваться на исследованиях, дизайн которых подразумевает оценку не только госпитального этапа лечения, но и отдаленных исходов для улучшения результатов профилактики, лечения и прогноза пациентов. В данной работе представлен анализ фрагмента объединенной когорты регистров АКТИВ и АКТИВ 2, включающих в себя периковидный и постковидный этапы лечения и наблюдения.

## ЦЕЛЬ ИССЛЕДОВАНИЯ

Изучить взаимосвязь НУО у пациентов с COVID-19 с летальностью, течением инфекции и отдаленными последствиями, а также выявить факторы риска неблагоприятного течения заболевания.

## МАТЕРИАЛЫ И МЕТОДЫ

Регистры АКТИВ и АКТИВ 2 — многоцентровые неинтервенционные регистры реальной клинической практики, которые включали в себя пациентов, перенесших COVID-19 в период с 29.06.2020 г. по 29.11.2020 г. (АКТИВ) и с 01.10.2020 г. по 30.03.2021 г. (АКТИВ 2).

Регистр АКТИВ состоит из двух непересекающихся ветвей — амбулаторной и госпитальной. В обеих ветвях исследования было предусмотрено 6 визитов: визит включения, визит на 7–12-е сутки, визит исхода (выписка/госпитализация/смерть и т.д.) и 3 визита спустя 3, 6 и 12 мес после выписки из стационара. В регистре АКТИВ 2 учитывались данные только госпитализированных пациентов и было предусмотрено 3 визита: визит включения, визит на 7–12-е сутки, визит исхода (выписка/госпитализация/смерть и т.д.).

Раннее были опубликованы дизайн, обоснование и статистический анализ исследований [11]. Нозологический диагноз устанавливался на основании критериев МКБ-10.

Всего в субанализ были включены 6396 пациентов из регистра АКТИВ и 2968 пациентов — из АКТИВ 2. В рамках проводимого субанализа выделены 3 группы пациентов: группа 1 — лица без НУО, которую составили 6606 больных (70,5%); группа 2 — пациенты с ВВГ (включая новые случаи гипергликемии и предсуществующий недиагностированный СД2), в которую вошли 1073 (11,6%) человека; группа 3 — пациенты с СД2 — 1611 (17,3%) человек (табл. 1). Необходимо подчеркнуть, что в группе 2 не представлялось возможным отделить ранее не диагностированный СД2 от новых случаев гипергликемии ввиду наблюдательного характера исследований АКТИВ и АКТИВ 2 и отсутствия данных по уровню гликированного гемоглобина у большинства пациентов. Гипергликемия диагностировалась при уровне глюкозы в плазме венозной крови ≥7,0 ммоль/л [5]. Важно отметить, что из субанализа были исключены несколько случаев СД 1 типа, а также лица, у которых в индивидуальные регистрационные карты не были занесены показатели гликемии, фиксируемые по принципу «если известно».

Средний возраст пациентов составил 59 [ 48; 68] лет, в группе пациентов с ВВГ — 63 [ 55; 71], в группе больных СД2 — 66 [59–73]. Согласно проанализированным данным, умерших пациентов было значимо больше в группе 2 (с ВВГ) — 109 (10,4%) случаев и в группе 3 (с СД2) — 177 (11,2%) случаев по сравнению с группой 1 (без НУО) — 545 (5,8%). Показатели, отражающие тяжесть течения COVID-19, а именно степень поражения легких на компьютерной томографии (КТ) 3–4, сатурация менее 94%, частота дыхательных движений более 22 в минуту, повышение температуры тела ≥38,6°С, чаще встречались в группах 2 и 3. Распространенность таких сопутствующих патологий, как артериальная гипертензия (АГ), фибрилляция предсердий, инсульт в анамнезе, ишемическая болезнь сердца (ИБС), хроническая сердечная недостаточность (ХСН), хроническая болезнь почек, анемия, была также значимо выше в группах 2 и 3 по сравнению с пациентами группы 1, не имеющих НУО, достигая достоверно самой высокой встречаемости в группе 3.

В настоящем субанализе объединенных регистров АКТИВ и АКТИВ 2 представлены результаты исследования пациентов с впервые выявленной и предсуществующей гипергликемией с оценкой влияния НУО на течение и исходы НКИ.

Статистический анализ

Для анализа данных использовалась программа IBM SPSS STATISTICS 26. Проверка на нормальность распределения проводилась с использованием критерия Колмогорова–Смирнова. Для анализа количественных данных с распределением, отличным от нормального, в 3 выборках использовался критерий Краскела–Уоллиса для независимых выборок, для оценки качественных параметров в 2 группах пациентов был применен хи-квадрат Пирсона или точный критерий Фишера в зависимости от минимального предполагаемого числа. Для признаков, имеющих статистически значимые различия, проводились оценка шансов с 95% доверительным интервалом (ДИ), а также определение меры связи между номинальными признаками. При анализе номинальных признаков в 3 группах использовались многопольные таблицы сопряженности с последующим проведением post-hoc анализа. Для сравнения номинальных признаков в 3 связанных совокупностях был применен критерий Кохрена. Методом бинарной логистической регрессии была разработана прогностическая модель для определения риска развития летального исхода в зависимости от возраста, уровня гликемии, наличия инфаркта миокарда (ИМ) и острого нарушения мозгового кровообращения в анамнезе, а также наличия сопутствующей патологии в виде ИБС. Отношение шансов (ОШ) с 95% ДИ для предикторов, оказывающих статистически значимое влияние на исход, было представлено в виде forest plot. Пороговое значение логистической функции Р было определено с помощью метода ROC-кривых.

## РЕЗУЛЬТАТЫ

Индекс массы тела (ИМТ) у пациентов в группе с ВВГ (группа 2) и в группе 3 с СД2 был значимо выше, чем в группе 1 (табл. 2). ИМТ менее 30 кг/м2 был в большей степени характерен для пациентов группы 1 — без НУО (р<0,001). Летальность в изучаемых группах имела статистически значимые различия (р<0,001): наибольшее количество смертельных исходов было отмечено в группе 2 (10,4% — 109 человек) и в группе 3 (11,2% — 177 человек), меньше всего умерших было в группе 1 (2,9% — 254 человека). Статистически значимые различия (р<0,001) наблюдались между изучаемыми группами в отношении степени КТ, отражающей объем поражения легких и тяжесть коронавирусной пневмонии. Выявлено, что КТ3 и КТ4 в 1,5–2 раза чаще встречались в группе 2 (183 (20,3%) и 47 (5,2%)) пациентов соответственно и в группе 3 (243 (17,6%) и 76 (5,5%)) пациентов соответственно, в то время как в группе больных без НУО значимо реже встречались КТ3 — у 574 (11,1%) и КТ4 — у 106 (2,0%). Уровень С-реактивного белка ≥50 мг/л, как и значение SpO2≤90%, статистически значимо чаще выявлялись в группе 2 и в группе 3 по сравнению с группой пациентов без НУО. Глюкокортикостероиды (ГКС) в большем количестве случаев назначались в группе 2, чем в остальных группах (р<0,001).

При сравнении летальности в зависимости от ВВГ или наличия СД2 получены статистически значимые различия (р<0,001). Так, шансы наступления летального исхода в группе пациентов с СД2 увеличивались в 2,48 раза (95% ДИ 2,05–3,00), а в группе больных с ВВГ — в 2,04 раза (95% ДИ 1,64–2,55). В то же время у пациентов без НУО (группа 1) по уровню летальности также имелись значимые различия, однако шансы наступления летального исхода у данной когорты, напротив, уменьшались в 2,94 раза (95% ДИ 0,28–0,40), что отражено на рисунке 1.

Особый интерес для изучения представляет когорта пациентов с НКИ среднетяжелого и тяжелого течения с сатурацией менее 93% (табл. 3). Летальность в данной когорте оказалась наиболее высокой и среди групп распределялась следующим образом: у пациентов с ВВГ (группа 2) она составила 21,4%, в группе с СД2 (группа 3) — 20,2%, а в группе 1 — 12,4%, что имело статистически значимые различия (р<0,001). В терапии ГКС больше всего нуждались пациенты с ВВГ — 41 (24,0%), тогда как в других группах данный вид терапии получали менее 14,1% пациентов (р=0,006).

Жалобы, предъявляемые на постгоспитальном этапе, — одна из серьезных причин снижения качества жизни пациентов после перенесенной НКИ и основание, которое позволило медицинскому сообществу сформулировать концепцию постковидного синдрома. В регистре АКТИВ наблюдение за пациентами после выписки проводилось через 3, 6 и 12 мес. За это время наиболее часто пациентов беспокоили: снижение вкуса, кашель, экспекторация мокроты, миалгии, торакалгии, сердцебиение, повышение артериального давления (АД), слабость, диарея, насморк, конъюнктивит, першение в горле, повышение температуры тела (табл. 4). Статистически значимо чаще встречались и дольше сохранялись в группах 2 и 3 жалобы на снижение вкусовых ощущений (робщ<0,001), кашель (робщ<0,001), мокроту (робщ<0,001), миалгии (робщ<0,001), торакалгии (робщ<0,001), сердцебиение (робщ<0,001), повышение АД (робщ<0,001) и слабость (робщ<0,001).

Сахароснижающую терапию в период НКИ получали все пациенты из группы 3 и 87% пациентов из группы 2. В соответствии с существующими подходами большой вклад в управление гипергликемией в период острой инфекции в обеих группах внесла инсулинотерапия [12], однако существенных различий в проводимой терапии не выявлено при тенденции к более широкому применению пероральных сахароснижающих препаратов (ПССП) в группе больных СД2 (табл. 5).

Через 12 мес в группе 2 только 18 человек (1,7%) получали антигипергликемические лекарственные средства, которые были представлены монотерапией ПССП. В группе 3 спустя год наблюдения отмечена трансформация сахароснижающей терапии: уже 72,6% находились на монотерапии ПССП, 18,9% — на двойной комбинированной терапии ПССП и только 8,5% пациентов управляли СД2 приемом базального инсулина ± ПССП.

В ходе исследования была разработана прогностическая модель для определения шанса развития летального исхода в зависимости от возраста, уровня гликемии, наличия ИБС, а также ИМ или инсульта в анамнезе. При проведении однофакторного анализа все вышеуказанные переменные имели статистически значимое влияние на вероятность наступления летального исхода. Важно отметить, что ИМТ>30 кг/м2 и АГ при поправке на другие факторы не оказывали достоверного влияния на смертность и достигали уровня значимости только при ассоциации с вышеперечисленными предикторами.

При проведении многофакторного анализа статистически значимое влияние на вероятность развития летального исхода также оказывали все исследуемые факторы. Характеристики каждого из них представлены в таблице 6.

Наблюдаемая зависимость описывается уравнением (1):

Р = 1 / (1 + e-z) * 100% (1)

z = - 7,95 + 0,06*Xвозраст + 0,12*Xгликемия+ + 0,48*XИМ+0,34*XИБС+0,76*Xинсульт,

где Р — вероятность наступления летального исхода (%); Xвозраст — возраст (лет); Xгликемия — уровень гликемии (ммоль/л); XИМ — наличие ИМ в анамнезе (0 — отсутствует, 1 — имеется); XИБС — наличие ишемической болезни сердца (0 — отсутствует, 1 — имеется); Xинсульт — наличие инсульта в анамнезе (0 — отсутствует, 1 — имеется).

Полученная регрессионная модель является статистически значимой (p<0,001). Исходя из значения коэффициента детерминации Найджелкерка, модель (1) учитывает 18,6% факторов, определяющих вероятность развития летального исхода.

В соответствии со значениями регрессионных коэффициентов, возраст, уровень гликемии, наличие ИМ, ИБС и инсульта в анамнезе имеют прямую связь с вероятностью развития летального исхода. Увеличение возраста на 1 год повышает шансы наступления летального исхода в 1,07 раза (95% ДИ 1,06–1,08), увеличение уровня гликемии на 1 ммоль/л приводит к повышению шансов наступления летального исхода в 1,12 раза (95% ДИ 1,1–1,15), наличие ИМ в анамнезе увеличивает вероятность наступления летального исхода в 1,62 раза (95% ДИ 1,22–2,16), ИБС — в 1,40 раза (95% ДИ 1,12–1,76), инсульта — в 2,14 раза (95% ДИ 1,58–2,90). На рисунке 2 сопоставлены значения ОШ (95% ДИ) наступления летального исхода для изучаемых факторов, вошедших в модель 1.

Пороговое значение логистической функции Р было определено с помощью метода анализа ROC-кривых. Полученная кривая представлена на рисунке 3.

Площадь под ROC-кривой соответствующей взаимосвязи прогноза летального исхода и значения логистической регрессионной функции составила 0,786±0,01 (95% ДИ 0,77–0,81). Значение логистической функции в точке cut-off составило 0,061. Значения функции, равные или превышающие данное значение, соответствовали высокому риску наступления летального исхода. При значениях функции менее 0,061 определялся низкий риск наступления летального исхода. Чувствительность и специфичность модели (1) при данном пороговом значении составили 72,2 и 68,5% соответственно.

**Table:** Таблица 1. Характеристика пациентов, включенных в регистры АКТИВ и АКТИВ 2

Показатель	Общая когортаn=9290	Группа 1(пациенты без НУО)n=6606	Группа 2(пациенты с ВВГ)n=1073	Группа 3(пациенты с СД2)n=1611	р1–3
Возраст	59[48–68]	58[46–67]	63[55–71]	66[59–73]	<0,001*Р1–2<0,001*Р1–3<0,001*Р2–3<0,001*
Женщины	4947 (53,2%)	3477 (52,2%)	533 (49,7%)	937 (58,2%)	<0,001*
Умершие	545 (5,9%)	259 (4,0%)	109 (10,4%)	177 (11,2%)	<0,001*
Избыточная масса тела	2921 (31,4%)	2135 (39,0%)	331 (36,5%)	455 (34,2%)	<0,001*
Ожирение 1 ст.	1700 (18,3%)	1044 (19,1%)	251 (27,6%)	405 (30,4%)
Ожирение 2 ст.	667 (7,2%)	379 (6,9%)	94 (10,4%)	194 (14,6%)
Ожирение 3 ст.	296 (3,2%)	147 (2,7%)	39 (4,3%)	110 (83%)
КТ1	3136 (33,8%)	2369 (45,5%)	291 (32,3%)	476 (34,5%)	<0,001*
КТ2	2563 (27,6%)	1695 (32,5%)	344 (38,2%)	524 (38,0%)
КТ3	1005 (10,8%)	579 (11,1%)	183 (20,3%)	243 (17,6%)
КТ4	231 (2,5%)	108 (2,1%)	47 (5,2%)	76 (5,5%)
SpO2 75–94%	2165 (23,3%)	1334 (29,1%)	289 (45,2%)	542 (51,8%)	<0,001*
SpO2≤75%	55 (0,6%)	21 (0,5%)	16 (2,5%)	18 (1,7%)
ЧДД 22–29	2312 (24,9%)	1448 (22,0%)	333 (31,4%)	531 (33,1%)	<0,001*
ЧДД более 30	178 (1,9%)	79 (1,2%)	33 (3,1%)	66 (4,1%)
Температура тела38,6–39,0°С	1633 (17,6%)	1114 (16,9%)	215 (20,2%)	304 (19,1%)	<0,001*
Температура тела>39,0°С	640 (6,9%)	431 (6,6%)	106 (10,0%)	103 (6,5%)
АГ	5289 (56,9%)	3242 (48,7%)	701 (65,3%)	1346 (83,6%)	<0,001*
Курение	475 (5,1%)	369 (5,5%)	37 (3,4%)	69 (4,3%)	0,004*
ФП	672 (7,2%)	387 (5,8%)	100 (9,3%)	185 (11,5%)	<0,001*
ИБС	2072 (22,3%)	1195 (18,0%)	273 (25,4%)	604 (37,5%)	<0,001*
ИМ в анамнезе	592 (6,4%)	324 (4,9%)	74 (6,9%)	194 (12,0%)	<0,001*
ХСН	1595 (17,2%)	888 (13,3%)	220 (20,5%)	487 (30,2%)	<0,001*
Инсульт в анамнезе	401 (4,3%)	226 (3,4%)	49 (4,6%)	126 (7,8%)	<0,001*
СД2	1611 (17,3%)	0	0	1611 (100%)	<0,001*
ХБП	716 (7,7%)	381 (5,7%)	93 (8,7%)	242 (15,0%)	<0,001*
ХОБЛ	408 (4,4%)	272 (4,1%)	49 (4,6%)	87 (5,4%)	0,065
БА	321 (3,5%)	219 (3,3%)	40 (3,7%)	62 (3,8%)	0,467
Рак в настоящее время	536 (5,8%)	372 (5,6%)	64 (6,0%)	100 (6,2%)	0,595
Анемия	1972 (21,2%)	1282 (21,1%)	248 (23,2%)	442 (28,4%)	<0,001*

**Table:** Таблица 2. Сравнительная характеристика пациентов в изучаемых группах в зависимости от наличия или отсутствия определенных признаков

Признак	Группа 1(пациенты без НУО)n=6606	Группа 2(пациенты с ВВГ)n=1073	Группа 3(пациенты с СД2)n=1611	р
ИМТ
Ме [Q1–Q3]	27,1 [ 24,2–30,5]	28,8 [ 25,6–32,7]	30,4 [ 27,0–34,6]	<0,001*
Менее 18,5	58 (1,1%)	7 (0,8%)	6 (0,5%)	<0,001*
18,5–24,9	1696 (31,2%)	186 (20,5%)	162 (12,2%)
25–29,9	2124 (39,1%)	331 (36,5%)	455 (34,2%)
30–34,9	1035 (19,1%)	251 (27,6%)	405 (30,4%)
35–39,9	376 (6,9%)	94 (10,4%)	194 (14,6%)
40 и более	144 (2,7%)	39 (4,3%)	110 (8,3%)
Летальность
Живы	6254 (96,1%)	944 (89,6%)	1406 (88,8%)	<0,001*
Умерли	254 (3,9%)	109 (10,4%)	177 (11,2%)
ОШ (95% ДИ)	0,34 (0,28–0,40)	2,04 (1,64–2,55)	2,48 (2,05–3,00)	
р	<0,001*	<0,001*	<0,001*
КТ
3	574 (11,1%)	183 (20,3%)	243 (17,6%)	<0,001*
4	106 (2,0%)	47 (5,2%)	76 (5,5%)
СРБ≥50 мг/л
Отсутствует	3796 (77,7%)	589 (68,0%)	808 (63,9%)	<0,001*
Имеется	1088 (22,3%)	277 (32,0%)	456 (36,1%)
ГКС
Не назначены	5735 (86,8%)	808 (75,3%)	1356 (84,2%)	<0,001*
Назначены	871 (13,2%)	265 (24,7%)	255 (15,8%)
Сатурация ≤90%
Отсутствует	4298 (94,4%)	554 (86,1%)	885 (84,6%)	<0,001*
Имеется	253 (5,6%)	86 (13,4%)	161 (15,4%)

**Figure:**
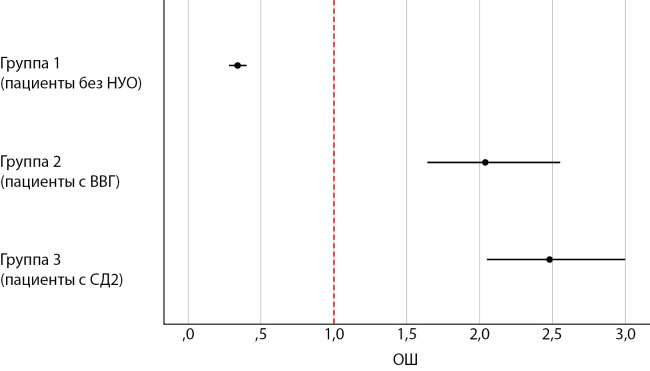
Рисунок 1. ОШ (95% ДИ) наступления летального исхода в зависимости от характера нарушения углеводного обмена.

**Table:** Таблица 3. Сравнительная характеристика пациентов в изучаемых группах с SpO2<93% в зависимости от летальности и назначения ГКС

Признак	Группа 1(пациенты без НУО)n=6606	Группа 2(пациенты с ВВГ)n=1073	Группа 3(пациенты с СД2)n=1611	р
Летальность
Выздоровели	829 (87,9%)	173 (78,6%)	343 (79,8%)	<0,001*
Умерли	114 (12,1%)	47 (21,4%)	87 (20,2%)
ГКС
Не назначены	638 (85,9%)	130 (76,0%)	319 (86,9%)	0,006*
Назначены	105 (14,1%)	41 (24,0%)	48 (13,1%)

**Table:** Таблица 4. Основные жалобы в изучаемых группах на постгоспитальном этапе через 3, 6 и 12 месяцев

Снижение/потеря вкуса	Группа 1 (пациенты без НУО)	82 (1,2%)	37 (0,6%)	15 (0,2%)	<0,001*р3–6<0,001*р3–12<0,001*р6–12=0,006*
Группа 2 (пациенты с ВВГ)	20 (1,9%)	10 (0,932%)	2 (0,186%)	<0,001*р3–6=0,008*р3–12<0,001*р6–12=0,109
Группа 3 (пациенты с СД2)	9 (0,559%)	6 (0,372%)	4 (0,248%)	0,895
p (между группами на каждом этапе)	0,009*	0,161	0,846	
Кашель	Группа 1 (пациенты без НУО)	209 (3,1%)	102 (1,5%)	51 (0,8%)	<0,001*р3–6=0,001р3–12<0,001*р6–12=0,003*
Группа 2 (пациенты с ВВГ)	38 (3,5%)	15 (1,4%)	9 (0,839%)	0,031*р3–6=0,064р3–12=0,011*р6–12=0,487
Группа 3 (пациенты с СД2)	55 (3,4%)	25 (1,6%)	13 (0,807%)	0,005*р3–6=0,021*р3–12=0,002*р6–12=0,401
р (между группами на каждом этапе)	0,066	0,752	0,643	
Мокрота	Группа 1 (пациенты без НУО)	47 (0,7%)	17 (0,3%)	8 (0,1%)	<0,001*р3–6=0,002*р3–12<0,001*р6–12=0,439
Группа 2 (пациенты с ВВГ)	11 (1,0%)	5 (0,466%)	2 (0,186%)	0,066
Группа 3 (пациенты с СД2)	9 (0,559%)	8 (0,497%)	6 (0,372%)	0,407
р (между группами на каждом этапе)	0,288	0,104	0,029*	
Миалгии	Группа 1 (пациенты без НУО)	80 (1,2%)	40 (0,6%)	20 (0,3%)	<0,001*р3–6<0,001*р3–12<0,001*р6–12 =0,312
Группа 2 (пациенты с ВВГ)	17 (1,6%)	11 (1,0%)	4 (0,373%)	0,047*р3–6=0,358р3–12=0,014*р6–12=0,126
Группа 3 (пациенты с СД2)	28 (1,7%)	9 (0,559%)	13 (0,807%)	0,296
р (между группами на каждом этапе)	0,015*	0,191	0,002*	
Торакалгии	Группа 1 (пациенты без НУО)	102 (1,5%)	56 (0,8%)	27 (0,4%)	0,013*р3–6=0,291р3–12=0,004*р6–12=0,064
Группа 2 (пациенты с ВВГ)	19 (1,8%)	14 (1,3%)	4 (0,373%)	0,025*р3–6=0,373р3–12=0,008*р6–12=0,075
Группа 3 (пациенты с СД2)	39 (2,4%)	33 (2,0%)	17 (1,1%)	0,349
р (между группами на каждом этапе)	0,001*	<0,001*	<0,001*	
Сердцебиение	Группа 1 (пациенты без НУО)	233 (3,5%)	140 (2,1%)	62 (0,9%)	<0,001*р3–6<0,001*р3–12<0,001*р6–12=0,012*
Группа 2 (пациенты с ВВГ)	47 (4,4%)	30 (2,8%)	18 (1,7%)	0,018*р3–6=0,099р3–120,005*р6–12=0,239
Группа 3 (пациенты с СД2)	65 (4,0%)	25 (1,6%)	22 (1,4%)	0,008*р3–6=0,003*р3–12=0,018*р6–12=0,584
р (между группами на каждом этапе)	0,005*	0,104	0,004*	
Повышение АД	Группа 1 (пациенты без НУО)	425 (6,4%)	369 (5,5%)	201 (3,0%)	0,047*р3–6=0,828р3–12=0,043*р6–12=0,025*
Группа 2 (пациенты с ВВГ)	74 (6,9%)	64 (6,0%)	33 (3,1%)	0,109
Группа 3 (пациенты с СД2)	126 (7,8%)	118 (7,3%)	48 (3,0%)	0,041*р3–6=0,593р3–12=0,061р6–12=0,016*
р (между группами на каждом этапе)	<0,001*	<0,001*	0,381	
Слабость	Группа 1 (пациенты без НУО)	699 (10,5%)	412 (6,2%)	209 (3,1%)	<0,001*р3–6<0,001*р3–12<0,001*р6–12<0,001*
Группа 2 (пациенты с ВВГ)	125 (11,6%)	65 (6,1%)	32 (3,0%)	<0,001*р3–6<0,001*р3–12<0,001*р6–12=0,017*
Группа 3 (пациенты с СД2)	193 (12,0%)	136 (8,4%)	74 (4,6%)	<0,001*р3–6=0,004*р3–12<0,001*р6–12=0,017*
р (между группами на каждом этапе)	<0,001*	<0,001*	<0,001*	
Диарея	Группа 1 (пациенты без НУО)	32 (0,5%)	9 (0,1%)	8 (0,1202%)	0,001*р3–6=0,001*р3–12=0,004*р6–12=0,655
Группа 2 (пациенты с ВВГ)	7 (0,652%)	2 (0,186%)	2 (0,186%)	0,607
Группа 3 (пациенты с СД2)	7 (0,435%)	3 (0,186%)	6 (0,372%)	0,497
р (между группами на каждом этапе)	0,568	0,731	0,029*	
Насморк	Группа 1 (пациенты без НУО)	27 (0,4%)	14 (0,2%)	6 (0,902%)	0,037*р3–6=0,136р3–12=0,011*р6–12=0,286
Группа 2 (пациенты с ВВГ)	5 (0,466%)	6 (0,559%)	1 (0,093%)	0,121
Группа 3 (пациенты с СД2)	6 (0,372%)	1 (0,062%)	3 (0,186%)	0,549
р (между группами на каждом этапе)	0,842	0,023*	0,388	
Конъюнктивит	Группа 1 (пациенты без НУО)	3 (0,045%)	0	2 (0,03%)	0,368
Группа 2 (пациенты с ВВГ)	0	0	0	-
Группа 3 (пациенты с СД2)	2 (0,124%)	0	0	-
р (между группами на каждом этапе)	0,240	-	0,710	
Першение	Группа 1 (пациенты без НУО)	40 (0,6%)	17 (0,3%)	9 (0,135%)	<0,001*р3–6<0,001*р3–12<0,001*р6–12=0,695
Группа 2 (пациенты с ВВГ)	7 (0,652%)	4 (0,373%)	1 (0,093%)	0,325
Группа 3 (пациенты с СД2)	7 (0,435%)	0	2 (0,124%)	0,066
р (между группами на каждом этапе)	0,812	0,099	0,951	
Повышение температуры тела	Группа 1 (пациенты без НУО)	51 (0,8%)	16 (0,2%)	5 (0,075%)	<0,001*р3–6<0,001*р3–12<0,001*р6–12=0,262
Группа 2 (пациенты с ВВГ)	5 (0,466%)	3 (0,280%)	2 (0,186%)	0,417
Группа 3 (пациенты с СД2)	4 (0,248%)	1 (0,062%)	4 (0,248%)	0,325
р (между группами на каждом этапе)	0,146	0,405	0,069	

**Table:** Таблица 5. Сахароснижающая терапия у пациентов групп 2 и 3 в период лечения COVID-19

Терапия	Группа 2(пациенты с ВВГ)n=1073	Группа 3(пациенты с СД2)n=1611	р
ПССП	323 (30,1%)	626 (38,9%)	<0,001*
Базальная инсулинотерапия ± ПССП	216 (20,1%)	368 (22,8%)	0,096
Базис-болюсная инсулинотерапия	395 (36,8%)	617 (38,3%)	0,437

**Table:** Таблица 6. Факторы, оказывающие влияние на риск наступления летального исхода по результатам многофакторного анализа

Предиктор	COR[ 95% ДИ]	р	АOR[ 95% ДИ]	Р
Увеличение возраста на 1 год	1,08 [ 1,07–1,09]	<0,001*	1,07 [ 1,06–1,08]	<0,001*
Увеличение уровня гликемии на 1 ммоль/л	1,14 [ 1,11–1,16]	<0,001*	1,12 [ 1,1–1,15]	<0,001*
ИМ в анамнезе	3,7 [ 2,92–4,69]	<0,001*	1,62 [ 1,22–2,16]	0,001*
ИБС	3,9 [ 3,28–4,66]	<0,001*	1,4 [ 1,12–1,76]	0,004*
Инсульт в анамнезе	4,80 [ 3,70 -6,22]	<0,001*	2,14 [ 1,58–2,90]	<0,001*

**Figure:**
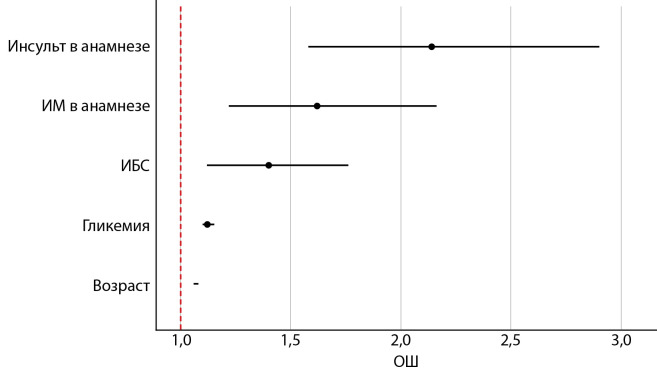
Рисунок 2. ОШ (95% ДИ) вероятности наступления летального исхода для изучаемых предикторов.

**Figure:**
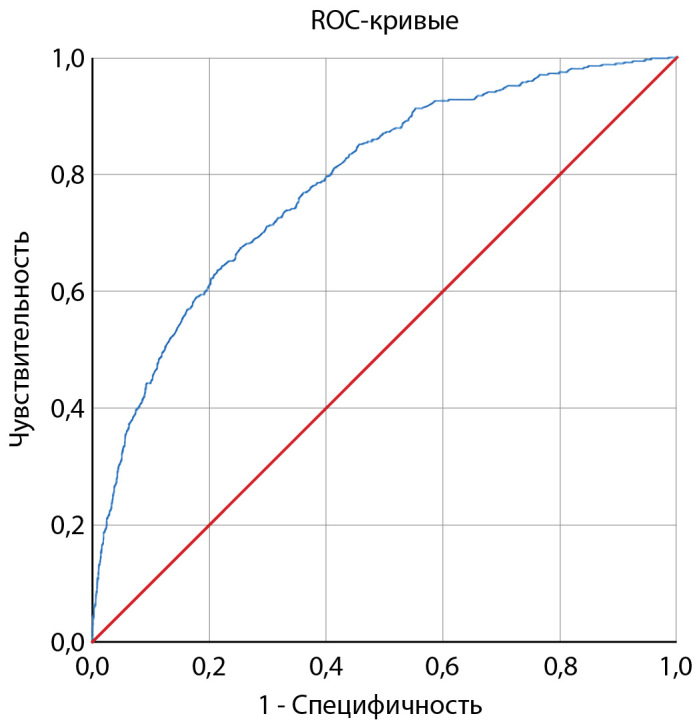
Рисунок 3. ROC-кривая, характеризующая зависимость вероятности наступления летального исхода от значений прогностической функции (1).

## ОБСУЖДЕНИЕ

Субанализ объединенных регистров реальной клинической практики АКТИВ и АКТИВ 2, включающих пациентов амбулаторного и госпитального этапов лечения COVID-19 и 12-месячного наблюдения в постковидном периоде, предназначен для оценки влияния гипергликемии на исходы НКИ. Это связано с особым значением, которое многими исследователями придается НУО у больных с COVID-19 как важнейшему фактору риска развития более тяжелого течения инфекции, а также неблагоприятных исходов [1][7][9][3].

В результате анализа данных установлено, что распространенность гипергликемии у пациентов с COVID-19 в выбранной когорте составляет 28,9%, из которых в 11,6% случаев обследованные имеют ВВГ без предшествующего анамнеза НУО, а 17,3% представлены больными с СД2.

Частота летальных исходов у пациентов с гипергликемией в субанализе составляет 10,6%, что значимо выше, чем у больных без НУО (3,9%), при этом летальность больных СД2 достоверно превышает таковую пациентов с ВВГ (11,2% vs 10,4%). Похожая тенденция отмечена и среди пациентов с дыхательной недостаточностью (SpO2<93%), у которых наличие ВВГ или СД2 определяло двукратное увеличение летальности.

Полученные данные, касающиеся уязвимости инфицированных SARS-CoV-2 пациентов с гипергликемией, соотносятся с результатами многочисленных отечественных и зарубежных исследований, в соответствии с которыми количество смертельных исходов среди лиц с СД также в 2–3 раза превышает таковое у лиц без СД. Так, во французском национальном исследовании CORONADO летальность пациентов с СД2 составила 20,6% [14], в США — 28,8% [15], в Великобритании — 30,1% [16], а по данным Федерального регистра сахарного диабета РФ — 15,2% [13].

По результатам субанализа объединенных регистров АКТИВ и АКТИВ 2 можно заключить, что наличие НУО ассоциировано с более тяжелым течением COVID-19. Это подтверждается значимо более высокой встречаемостью КТ3 и КТ4 и среди пациентов с СД2 и с ВВГ, а также, по данным клинико-лабораторных показателей, большей выраженностью иммуновоспалительного синдрома по сравнению с лицами без НУО.

Данная закономерность корреспондирует с зарубежными данными: в метаанализе 47 исследований было продемонстрировано, что СД связан с более выраженной тяжестью течения COVID-19 (OШ 2,20; 95% ДИ 1,69–2,86; p<0,00001) и летальностью (OШ 2,52; 95% ДИ 1,93–3,30; p<0,00001) [17]. Метаанализ 16 исследований показал, что пациенты с предсуществующим СД2 имели повышенный риск более тяжелого течения НКИ (COVID‐19) (ОШ 2,60; 95% ДИ 1,96–3,45; p=0,01) [18]. В объединенном анализе 33 исследований была обнаружена значимая связь СД2 с более тяжелым течением инфекционного процесса (ОШ 2,75; 95% ДИ 2,09–3,62; p<0,01) и с более высокой смертностью от COVID-19 с общим ОШ 1,90 (95% ДИ 1,37–2,64; p<0,01) [19].

Интересно, что вид НУО оказывает различное по выраженности влияние на летальность пациентов с COVID-19. Действительно, наличие СД2 в нашем исследовании увеличивало шансы наступления смертельного исхода в 2,48 раза, а ВВГ — только в 2,04. Напротив, отсутствие НУО уменьшало шансы наступления смерти в 2,94 раза. Ровно так же пациенты с ВВГ в большинстве случаев характеризовались более неблагоприятным течением COVID-19, чем лица без НУО, но не достигали по клинико-лабораторным показателям и объему поражения легких уровня больных СД2.

Это входит в некоторое противоречие с результатами многих исследователей, показавших, что ВВГ является фактором более неблагоприятного течения и прогноза COVID-19, чем известный СД2 [10][20–22]. По-видимому, подобные различия в нашем случае можно объяснить ограничениями наблюдательного дизайна исследования с невозможностью разделения недиагностированного СД2 со стрессовой и стероид-индуцированной гипергликемией.

Особый интерес вызывают результаты, которые продемонстрировали влияние периковидных НУО на течение отдаленного постковидного периода. В субанализе объединенных регистров АКТИВ и АКТИВ 2 были выявлены более высокая частота жалоб и их более длительное сохранение у пациентов с НУО в постковидном периоде. Так, через 12 мес после перенесенной инфекции экспекторация мокроты, торакалгия, сердцебиение, АГ, слабость и диарея значимо чаще встречались у больных СД2 по сравнению с пациентами без НУО. Эти же жалобы, за исключением торакалгии и слабости, более активно предъявляли лица с ВВГ (но реже, чем больные СД2). Полученные данные позволят скорректировать программу реабилитации в постковидном периоде, поскольку пациентов с ВВГ и СД2 следует отнести к группе риска в отношении более длительных и устойчивых жалоб после инфекции [23]. К сожалению, из-за низкого отклика оказалось сложно отследить постковидную летальность, однако данные других исследований показывают, что СД являлся одним из ранних независимых предикторов 90-дневной смертности в выборке из 4643 пациентов с тяжелым течением COVID-19 [24], наличие СД2 у пациентов старше 60 лет увеличивало шанс смерти через 3 мес (ОШ 2,55; 95% ДИ 1,16–5,61; p=0,016) [25], а анализ более 100 тыс. госпитализированных пациентов с COVID-19 в США показал, что через 6 мес риск повторной госпитализации при наличии СД увеличивается на 20% [26].

Существенным результатом настоящего исследования является регистрация высокой частоты сопутствующих заболеваний у пациентов с НУО. Многофакторный анализ показал, что каждое из таких коморбидных состояний, как ИБС, ИМ и инсульт в анамнезе, приводит к существенному увеличению вероятности развития летального исхода в 1,4, в 1,6 и в 2,1 раза соответственно. Интересно, что ожирение, а также АГ, которые, по данным различных исследований, повышали риск наступления летального исхода [7][27], в нашем субанализе не демонстрировали самостоятельного влияния, если не были ассоциированы с вышеперечисленными заболеваниями.

Значение коморбидной патологии у больных СД2 было показано в ранее опубликованных результатах исследования по данным регистра АКТИВ. СД в сочетании с ожирением и сердечно-сосудистыми заболеваниями (ССЗ) приводил к значимому увеличению вероятности летального исхода в остром периоде НКИ на госпитальном этапе (ОШ 2,24; 95% ДИ 1,59–3,15; p<0,01 для всех пациентов и ОШ 2,51; 95% Д: 1,69–3,72; p<0,01 для лиц старше 60 лет). Сочетания АГ + ожирение + СД и АГ + ИБС + ХСН + СД также значимо увеличивали шанс летального исхода (ОШ 2,17; ДИ 1,53–3,08; p<0,01 и ОШ 4,21; 95 ДИ 2,78–6,38; p<0,01 соответственно) [3][28]. Интересно, что отмечалось повышение среднего уровня глюкозы в зависимости от наличия и количества коморбидных заболеваний: 5,44 ммоль/л для пациентов без ССЗ, 6,07 ммоль/л для пациентов с наличием одного ССЗ, 7,1 ммоль/л для пациентов с наличием 2 и 3 ССЗ и 7,79 ммоль/л для пациентов с наличием 4 ССЗ и более (p<0,01) [3].

Одной из важных задач настоящего исследования явилась оценка у пациентов с COVID-19 частоты новых случаев НУО, характера этих нарушений и их стойкости. По данным субанализа, ВВГ в периковидный период (11,6% случаев) реализовалась через 12 мес в 1,7% новых случаев СД2, который управлялся ПССП. Это свидетельствует о транзиторном характере «периковидной гипергликемии», но с учетом ее негативного влияния на течение и исходы инфекции требует ранней диагностики и серьезных усилий по своевременной коррекции.

К новым случаям СД в реконвалесценции COVID-19 приковано внимание большого количества исследователей. Различные тяжесть COVID-19, гендерный и этнический состав, разные штаммы SARS-CoV-2, доминирующие в тот или иной период пандемии, а также неунифицированные медикаментозные подходы по применению ГКС определяют чрезвычайно гетерогенные когорты для изучения частоты новых случаев СД [29]. Видимо, именно этим объясняется незначительное количество работ, которые бы проанализировали исходы ВВГ в отдаленном постковидном периоде, и именно это формирует острую потребность в таких исследованиях. Особняком здесь стоит убедительная работа W. Rathmann и соавт. [30], которые при оценке базы из 8,8 млн пациентов, сопоставив новые случаи СД после COVID-19 с таковыми после острого респираторного заболевания, установили на 28% более высокую частоту встречаемости после COVID-19 СД2, новые случаи которого были выявлены и в нашей работе.

В литературе имеется немало гипотез о возможных путях патологического взаимодействия SARS-CoV-2 и НУО. Точные механизмы, лежащие в их основе, окончательно неизвестны, но, вероятно, имеется целый ряд сложных взаимосвязей, включая непосредственное повреждение клеток поджелудочной железы со снижением секреции инсулина, воздействие острого воспалительного процесса с повышением инсулинорезистентности, стрессовую гипергликемию и негативное влияние терапии ГКС, приводящей к стероид-индуцированной гипергликемии (рис. 4).

Таким образом, взаимодействие между COVID-19 и СД можно охарактеризовать как двунаправленную связь между инфекционным процессом и НУО, осмысление которой и своевременные профилактические мероприятия позволят минимизировать негативное влияние этого опасного «тандема» на прогноз пациента не только в эту пандемию, но и в последующие.

Ограничения исследования

АКТИВ и АКТИВ 2 являются регистрами реальной клинической практики. Данные для некоторых переменных вводились по принципу «если известно» и не были обязательны для заполнения. В связи с этим существует некоторая потеря данных на этапе их ввода врачами-исследователями, и точность информации, полученной при телефонном разговоре, может быть ограничена. Также регистры заполнялись на разных этапах изменения федеральных клинических рекомендаций по ведению пациентов с НКИ (изменения касались в основном ведения и терапии пациентов с НКИ). Необходимо учесть, что в начале пандемии (весна и лето 2020 г.) фактическое количество госпитализаций по причине инфицирования SARS-CoV-2 было выше, чем необходимое по прямым медицинским показаниям, ввиду недостаточного количества информации о данной патологии, в связи с чем можно считать, что в регистре представлены пациенты с различной степенью тяжести COVID-19.

**Figure:**
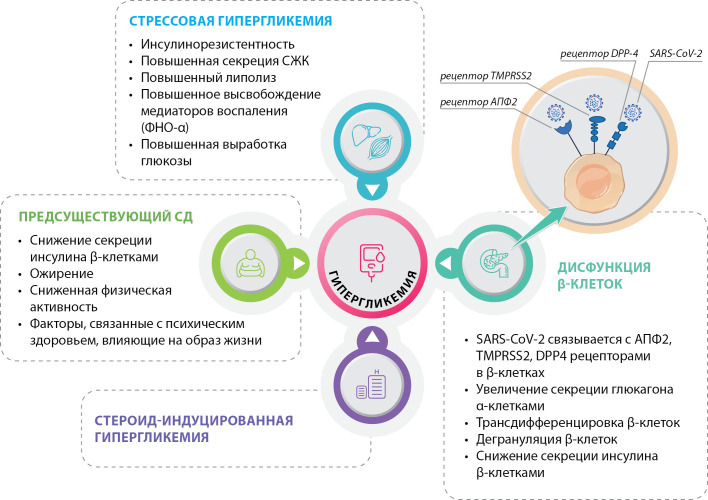
Рисунок 4. Возможные механизмы развития впервые выявленной гипергликемии/сахарного диабета у пациентов с COVID-19. Адаптировано [31]. Примечание. АПФ2 — ангиотензинпревращающий фермент 2; TMPRSS2 — трансмембранная серинпротеаза 2 типа; DPP-4 — дипептидилпептидаза-4 типа; СЖК — свободные жирные кислоты; ФНО-α — фактор некроза опухоли-альфа.

## ЗАКЛЮЧЕНИЕ

На сегодняшний день представленный субанализ объединенных регистров АКТИВ и АКТИВ 2 является одним из самых масштабных — более 9 тысяч пациентов — исследованием, которое посвящено изучению влияния НУО на исходы COVID-19. Важной его особенностью следует считать оценку не только влияния ВВГ и СД2 на течение инфекции и летальность в периковидный период, но и исследование отдаленных исходов через 12 мес после COVID-19.

В результате исследования выявлено, что НУО приводят к ухудшению течения НКИ в виде более значимого поражения легочной ткани и дыхательной недостаточности, более выраженному иммуновоспалительному синдрому. Продемонстрировано, что периковидная гипергликемия (ВВГ+СД2) на 20% повышает шансы летального исхода, а его предикторами являются такие коморбидные состояния, как ИБС, ИМ или инсульт в анамнезе, более старший возраст.

Через год после перенесенной инфекции у пациентов с СД2 и ВВГ чаще сохраняются жалобы, характерные для постковидного синдрома, а ВВГ после острого периода заболевания нивелируется в 1,7% новых случаев пациентов с СД2.

## ДОПОЛНИТЕЛЬНАЯ ИНФОРМАЦИЯ

Источники финансирования. Авторы декларируют отсутствие внешнего финансирования для проведения исследования и публикации статьи.

Конфликт интересов. Авторы декларируют отсутствие явных и потенциальных конфликтов интересов, связанных с публикацией настоящей статьи.

Участие авторов. Салухов В.В. — концепция и дизайн исследования, получение и интерпретация результатов, написание рукописи; Арутюнов Г.П. — концепция и дизайн исследования, получение и интерпретация результатов; Тарловская Е.И. — концепция и дизайн исследования, получение и интерпретация результатов; Батлук Т.И. — получение и интерпретация результатов, написание рукописи, Башкинов Р.А. — получение и интерпретация результатов, написание рукописи; Самусь И.В. — статистический анализ данных, интерпретация результатов, написание рукописи; Мельников Е.С. — получение и интерпретация результатов; Трубникова М.А. — получение и интерпретация результатов; Арутюнов А.Г. — концепция и дизайн исследования, получение и интерпретация результатов, внесение правок. Все авторы одобрили финальную версию статьи перед публикацией, выразили согласие нести ответственность за все аспекты работы, подразумевающую надлежащее изучение и решение вопросов, связанных с точностью или добросовестностью любой части работы.
